# Isolation and Characterization of Anaerobic Bacteria for Symbiotic Recycling of Uric Acid Nitrogen in the Gut of Various Termites

**DOI:** 10.1264/jsme2.ME11325

**Published:** 2012-02-04

**Authors:** Arunee Thong-On, Katsuyuki Suzuki, Satoko Noda, Jun-ichi Inoue, Susumu Kajiwara, Moriya Ohkuma

**Affiliations:** 1 Microbe Division/Japan Collection of Microorganisms, RIKEN BioResource Center, Hirosawa 2–1, Wako, Saitama 351–0198, Japan; 2 Graduate School of Bioscience and Biotechnology, Tokyo Institute of Technology, Nagatsuda-cho 4259, Midori-ku, Yokohama, Kanagawa 226–8501, Japan; 3 Interdisciplinary Graduate School of Medicine and Engineering, University of Yamanashi, Takeda 4–3–11, Kofu, Yamanashi 400–8511, Japan; 4 Synaptech Co. Ltd., Ohte 1–2–37–C–105, Kofu, Yamanashi 400–0015, Japan

**Keywords:** symbiosis, uric acid, nitrogen recycling, anaerobic bacteria, termite

## Abstract

Recycling of the nitrogenous waste uric acid (UA) of wood-feeding termites by their gut bacteria is one of the significant aspects of symbiosis for the conservation of nitrogen sources. Diverse anaerobic UA-degrading bacteria comprising 16 species were isolated from the gut of eight termite species, and were assigned to *Clostridia*, *Enterobacteriaceae*, and low G+C Gram-positive cocci. UA-degrading *Clostridia* had never been isolated from termite guts. UA-degrading ability was sporadically distributed among phylogenetically various culturable anaerobic bacteria from termite guts. A strain of *Clostridium* sp., which was commonly isolated from three termite species and represented a probable new species in cluster XIVa of clostridia, utilized UA as a nitrogen source but not as a sole carbon and energy source. This feature is in clear contrast to that of well-studied purinolytic clostridia or previously isolated UA degraders from termite guts, which also utilize UA as a sole carbon and energy source. Ammonia is the major nitrogenous product of UA degradation. Various purines stimulated the growth of this strain when added to an otherwise growth-limiting, nitrogen poor medium. The bacterial species involved the recycling of UA nitrogen in the gut microbial community of termites are more diverse in terms of both taxonomy and nutritional physiology than previously recognized.

Uric acid (UA) is a major nitrogenous waste excreted from animals such as terrestrial insects, birds, and certain reptiles (57 and references therein). Because of its poor solubility in water (only 60 mg L^−1^ at 20°C), its excretion as a nontoxic solid minimizes water loss. Not merely regarded as nitrogenous waste, UA is apparently utilized as a nitrogen source or metabolic reserve in some insects, particularly those existing on a nitrogen-poor diet (*e.g.* 6, 10, 18, 44). Nitrogen acquisition is a primary concern of insects feeding on diets mostly composed of plant materials.

Wood-feeding termites are one of the best-studied insects, and their strategy to recycle UA nitrogen, and therefore to conserve combined nitrogen, is clearly elucidated (41–44). Wood-feeding termites store UA in their fat bodies and transfer it via Malpighian tubules to the hindgut, where gut microbiota recycle UA nitrogen. Termite tissues lack uricase or any UA utilizing activity, whereas gut microbiota degrade UA and consequently the feces of termites contain only small amounts of UA (43, 44). UA degradation in the termite gut is an anaerobic process responsible primarily for gut bacteria (44). It is estimated that an amount of uric acid nitrogen equivalent to 30% of the total nitrogen in an average termite colony may be recycled or redistributed annually through the action of gut uricolytic bacteria (3, 44). Besides the decomposition and utilization of cellulose and acquisition of new nitrogen by fixation of atmospheric N_2_, the recycling of UA nitrogen is an important and significant aspect of the symbiosis with gut microbiota (3, 4). Termite-gut microbiota form a complex, highly structured, but stable microbial community comprising largely yet-uncultivated, novel and diverse species (9, 32, 39).

Uricolytic bacteria have been isolated from the termite gut, and well studied physiologically (41, 42). The isolates are assigned to any of *Streptococcus* sp. (according to the original description), *Sebaldella* (formerly *Bacteroides*) *termitidis*, and *Citrobacter* sp. Despite the repeated extensive isolation, however, only a single species of the subterranean termite *Reticulitermes flavipes* has been investigated. There are more than 270 termite genera and a marked difference in gut microbiota has been disclosed among genera (11, 56); therefore, studies on the diversity of UA-degrading bacteria in various termites and characterization of their nutritional physiology are of particular significance to understand the nature of the gut microbial community and the symbiosis with host termites. In this study, we isolated and identified anaerobic uricolytic bacteria from eight various termite species. An isolate in the genus *Clostridium* (designated strain NkU-1) was further investigated for its properties of UA utilization.

## Materials and Methods

### Isolation and taxonomic characterization

Termite species used in this study are shown in Table 1. Uricolytic bacteria in the gut of termites were isolated according to basically the same methods described by Potrikus and Breznak (41). Worker termites were collected directly from the infested wood, and after washing the surface of the body with distilled water, their gut was removed from the body and squeezed into 0.4% (w/v) NaCl solution under anaerobic conditions. After appropriate dilution with the same solution, the gut homogenate was inoculated onto the top layer of the isolation plate (see below). The composition of culture media is described as a percentage (w/v) in this study unless indicated otherwise. The isolation plate (BHIU) comprised double-layer agar of brain-heart infusion (Difco) 3.7%, cystein-HCl 0.05%, and resazulin 10^−4^% containing 1.0 and 0.1% of UA and 0.6 and 1.5% agar in the top and bottom layers, respectively. The inoculated plate was anaerobically incubated at 25°C for 14 days. The colonies surrounded by a clear zone in the otherwise opaque medium were counted as presumptive uricolytic bacteria. Anaerobic manipulation and cultivation were performed in an anaerobic chamber (Bactron; Amco) with an O_2_-free atmosphere of N_2_/H_2_/CO_2_ (80/10/10, v/v/v). In the case of the termite *Reticulitermes speratus*, we applied a different method to identify UA-degrading bacteria; anaerobic bacteria were first isolated in medium that did not contain UA, as described previously (37), and after identification, representatives were examined for UA degradation by inoculating the isolates onto the BHIU plate.

Clear zone-forming uricolytic bacteria were isolated and purified after successive passages on streak plates; their purity was confirmed by microscopic observation including Gram staining of cells and direct sequencing of PCR product of the 16S rRNA gene. The 16S rRNA gene was amplified, purified, and sequenced partially with 750R primer as described previously (35, 38). The entire sequence of the amplified DNA was determined in some strains using primers described previously (33, 35, 38). Taxonomic assignment of the strains was then performed based on the 16S rRNA sequence using the Naïve Bayesian classifier (ver. 2.2) (58) provided by the Ribosomal Database Project (http://rdp.cme.msu.edu/index.jsp), with an 80% confidence threshold. For detailed comparisons, the quality-checked sequences of type strains in “The All-Species Living Tree” project (61) were obtained from the SILVA rRNA database project (http://www.arb-silva.de/) and used. Phylogenetic analysis with the maximum likelihood method was performed with RAxML 7.2.6 (53) using the GTRGAMMAI model and default settings. The classification was confirmed by Gram staining, cell shape, aerobic growth, catalase and oxidase activity, and oxidative-fermentative (O-F) test when the strain grew aerobically. Representative strains of the uricolytic isolates were deposited in the Japan Collection of Microorganisms (JCM) and their accession numbers are shown in Table 2. The DNA sequences for representative strains of the identified species were deposited in the DDBJ/EMBL/GenBank database under accession numbers AB673451 to AB673466.

PYG medium (8) was used for the culture of strain NkU-1 for standard taxonomic characterization. Utilization of sugars and other characteristics were examined using a commercially available identification kit, API20A (BioMerieux), according to the manufacturer’s instructions. The G+C content of DNA was determined by a previously reported method (54). Fatty acid analysis was conducted as described previously (14). Type strains of *Clostridium* spp., *C. sphenoides* JCM 1415^T^, *C. symbiosum* JCM 1297^T^, *C. celerecrescens* DSM 5628^T^, *C. aerotolerans* DSM 5434^T^, and *C. xylanolyticum* DSM 6555^T^, which were obtained from JCM and German Collection of Microorganisms and Cell Cultures, were used as references mainly for taxonomic studies. The UA degradation ability of these type strains was examined as described above using the BHIU plate. Previously isolated strains from *R. flavipes*(41) were also used as references for UA degradation.

### Nutritional studies

Most nutritional experiments of strain NkU-1 used growth-limiting Y basal medium, which comprised 0.01% yeast extract (Difco), 2.5% (v/v) 40× salt solution, 5% (v/v) 20× KP buffer, 0.4% (v/v) vitamin solution, and 10^−4^% resazurin-Na; 40× salt solution comprised 2% MgSO_4_·7H_2_O, 0.04% NaCl, 0.06% FeSO_4_·7H_2_O, 0.26% CaCl_2_·2H_2_O, and Na-thioglycolate; 20x KP buffer comprised 5.24% KH_2_PO_4_ and 10.71% K_2_HPO_4_; and vitamin solution contained 0.05% thiamine, 0.05% riboflavin, 0.05% pyridoxine-HCl, 0.05% calcium pantothenate, 0.01% nicotinic acid, 0.01% biotin, 0.01% folic acid, and 0.01% *p*-aminobenzoic acid. GY medium was supplemented by 1% glucose in Y medium. TYU medium comprising 1% tryptone (Difco), 0.1% yeast extract, 0.1% UA, 2.5% (v/v) 40× salt solution, and 0.4% (v/v) vitamin solution was also used for the culture of several strains to examine UA degradation. In the case of strain Cd23, GY instead of TYU medium was used for this purpose. UA was solubilized as 2% solution in 0.5 N NaOH, filter sterilized and added at an appropriate final concentraion, and before inoculation, a predetermined amount of sterile 0.5 N HCl was added to neutralize the medium. Purines to be added to the medium were solubilized as described above for UA and in the previous report (41). UA, purine-related compounds, and other chemicals were obtained from Nacalai Tesque (Kyoto, Japan) or various commercial sources. The culture in a glass bottle was sealed with a butyl rubber stopper in the anaerobic chamber and incubated at 37°C for three days with gentle shaking unless indicated otherwise.

In liquid culture, cell growth was measured by optical density (OD) at 660 nm or by the protein amount using Bio-Rad Protein Assay Reagent after adding NaOH (final 1N) and boiling the culture. UA was measured spectrophotometrically by the difference in its specific absorbance at 292 nm before and after treatment with pig liver uricase (Toyobo). NH_3_ was determined enzymatically by NH_3_-dependent oxidation of NADPH (measured by absorbance at 340 nm) with beef liver glutamate dehydrogenase (Roche Applied Science). UA and NH_3_ in growth media were assayed after removal of cells by centrifugation. All measurements were performed in duplicate and mean values are shown.

## Results and Discussion

### Diversity of UA-degrading bacteria

Anaerobic uricolytic bacteria were successfully detected on the BHIU plate from eight termite species at rates ranging 0.1 to 48.6% CFU (Table 1). They corresponded to the population level of 2.1×10^3^ to 4.0×10^4^ cells per gut except in the case of *Neotermes koshunensis* (1.1×10^6^ cells per gut). Because of the low culturability of gut bacteria (0.6% to 13% of total gut bacteria (37, 50)), isolated uricolytic bacteria seem to represent mere minor populations in the guts. Nevertheless, diverse uricolytic bacteria were identified in this study (see below). A soil-feeding termite *Pericapritermes nitobei* (Termitidae) was also examined, but uricolytic bacteria were not obtained, probably because its food (soil organic matter) is rich in nitrogenous compounds and recycling of UA is less significant in this termite.

The uricolytic isolates were taxonomically classified, and 16 different species were identified (Table 2). They were classified into any of the three bacterial groups *Clostridia*, *Enterobacteriaceae* (*Gammaproteobacteria*), and low G+C Gram-positive cocci (*Bacilli*). To our knowledge, this is the first report for the isolation of uricolytic bacteria in *Clostridia* from termite guts, although a variety of *Clostridia* strains have been isolated (for reviews, see references 3, 4). Bacteria related to *S. termitidis* (belonging to *Fusobacteria*) could not be isolated in this study.

Strains belonging to *Clostridia* occurred in six of the eight examined termite species (Table 1). On the basis of the 16S rRNA gene sequence, they were classified into five species in any of the clusters XIVa, XI, and I of clostridia, as defined by Collines *et al.*(5) (Table 2). Species represented by strain NkU-1 (cluster XIVa) were common to three termite species and occurred frequently among uricolytic isolates from *Hodotermopsis sjoestedti*. Species represented by strain Cd6 (cluster XIVa), which occurred commonly in two termites and frequently among uricolytic isolates from *Nasutitermes takasagoensis*, were closely related to *C. sphenoides* and *C. celerecrescens* (each 99.9% identity). Indeed, type strains of these two *Clostridium* species degraded UA in the BHIU plate medium as much as strains NkU-1 and Cd6 (determined by clear zone formation), whereas those of *C. xylanolyticum* and *C. aerotorelance* did not (data not shown). All of the isolates in *Clostridia* were distantly related to well-studied UA-degrading *Clostridium* species, *C. acidiurici*, *C. purinolyticum* (both belonging to cluster XII), and *C. cylindrosporum* (forms a unique lineage near cluster I or II) (45, 49, 57). From avian ceca, phenotypically diverse uricolytic bacteria are isolated and many apparently belong to *Clostridia*(1, 48), although most were distinct from the isolates in this study and classified into the genus *Eubacterium* or *Peptostreptococcus*.

From *R. speratus*, various anaerobic bacteria affiliated to the genera *Enterobacter*, *Clostridium*, *Lactococcus*, *Bacillus*, and *Bacteroides* were isolated in addition to previously reported *Disgonomonas* sp. (37). Nevertheless, only *Enterobacter* sp. (strain RsN-1) could degrade UA on the BHIU plate. The isolates in *Clostridium* and *Lactococcus* from *R. speratus* corresponded to completely different species from the uricolytic strains from other termites. Furthermore, *Lactococcus* spp. are those of major culturable bacteria in termite guts, but only a few among numerous isolates have demonstrated UA-degrading ability (2, 41, 50); therefore, UA degraders were sporadically distributed in phylogenetically diverse culturable anaerobic bacteria even in the same genus (*i.e. Lactococcus* or *Clostridium*).

### UA utilization and taxonomy of Clostridium sp. strain NkU-1

The degradation of UA was initially examined in liquid culture in several strains. *Clostridium* sp. strain NkU-1, its closely related three strains from the same termite, and *Lactococcus* sp. strain Cd31 degraded UA almost completely, whereas *Enterococcus* sp. strain Cd23, *Enterobacter* sp. strains GfU-1, and its close relative isolated from the same termite degraded UA only partially (56%, 31%, and 12% of added UA after 4 days culture, respectively). Incomplete UA degradation is quite a similar phenomenon to those reported previously for isolates from the termite gut (41, 42). Indeed, in the case of strain UAD-1 (originally described as group N *Streptococcus* sp. (41) but our preliminary analysis indicated that it belonged to the genus *Enterococcus*), incomplete degradation of UA has been shown unless formate or a formicogenic substrate as a reductant is present in the medium (42). The previously isolated strain of *Citrobacter* sp. degrades only a small amount of UA in liquid culture, although it forms a prominent clear zone in plate medium and the degradation of UA on the plate is verified (41, 42). Strain NkU-1 represents one of the most common uricolytic bacteria, it degraded UA well in liquid culture, and the physiology of related species from the termite gut has not been characterized so far. Thus, the following taxonomic and nutritional investigations focused on this strain.

The cells of strain NkU-1 were motile, peritrichous, straight rods, 2.0–4.0×0.8–1.0 μm, and often occurred in short chains in the log growth phase. Spores were oval and terminal or subterminal. A major fermentation product from glucose (PYG medium) was acetate (73%), but formate, propionate, and butylate were also detected (10.1–4.8%). The temperature for optimum growth was 35°C and optimum pH for growth was 7.2; the specific growth rate was 0.71 μ per hour under the optimal condition. Catalase, oxidase, and urease were negative. Indol was produced. Strain NkU-1 utilized a variety of carbohydrates and produced acids from glucose, mannitol, lactose, saccharose, maltose, salicin, xylose, arabinose, gelatin, esculin, glycerol, cellobiose, mannose, melezitose, raffinose, rhamnose, and trehalose, but not from sorbitol.

**Fig. 1:**
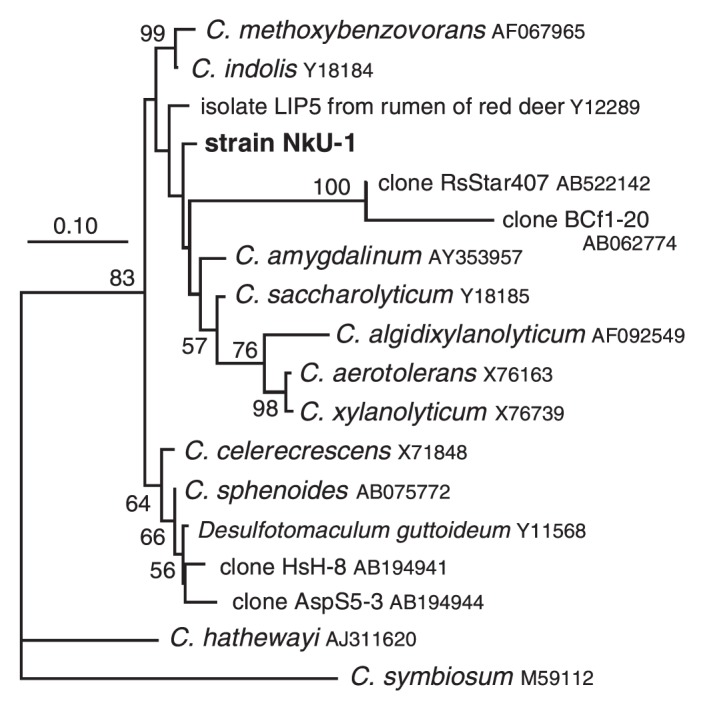
A maximum likelihood tree showing the phylogenetic position of strain NkU-1 in the clostridia cluster XIVa. The tree was reconstructed with 1,345 unambiguously aligned nucleotide sequences of 16S rRNA gene using *C. symbiosum* as an outgroup. In addition to type strains and isolate LIP5 (17), the analysis included sequences of four clones from termite guts: RsStar407 from *R. santonensis*(16), BCf1–20 from *C. formosanus*(51), HsH-8 from *H. sjoestedti*(30), and AspS5-3 from *Archotermopsis* sp. (30). The accession number for each sequence is shown. Percent bootstrap value in 1,000 replicates when above 50% is shown at each node. Scale bar represents 0.10 substitutions per position.

Based on 16S rRNA gene analysis, the closest relatives of strain NkU-1 were *Clostridium saccharolyticum* (98.9% identity) and *Clostridium amygdalium* (98.8%), although their relationships were not well resolved in the phylogenetic tree (Fig. 1). *C. saccharolyticum* and *C. amygdalium* showed 98.9% identity. The sequence of strain NkU-1 showed less than 98.6% identity to the other species. *C. saccharolyticum* shows similar characteristics, utilizing a variety of carbohydrates to strain NkU-1, but differs in that it is negative in Gram staining, is non-motile, and has no flagella (26). *C. amygdalium* is moderately thermophilic (optimum temperature 45°C) and shows a narrower spectrum of carbohydrate utilization (45). In addition, G+C content of *C. saccharolyticum* and *C. amygdalium* (28 and 32%, respectively) is lower than that of strain NkU-1 (41.1±1.2%; *n*=4); therefore, strain NkU-1 likely corresponds to a novel species in the clostridia cluster XIVa, although further study, such as measuring DNA relatedness, is necessary.

In the phylogenetic tree (Fig. 1), two clone sequences, RsStar407 and BCf1–20, from the gut of the termites *Reticulitermes santonensis*(16) and *C. fromosanus*(51), respectively, nested very closely to strain NkU-1, although the branch leading to these sequences was long and the sequence identities to strain NkU-1 were relatively low (95.7% and 94.2%, respectively). They are probably phylogenetically closely related to strain NkU-1, but rapidly evolving species inhabit these termites. An isolate from the rumen of red deer (17) is also closely related to strain NkU-1 with 98.7% sequence identity.

**Fig. 2:**
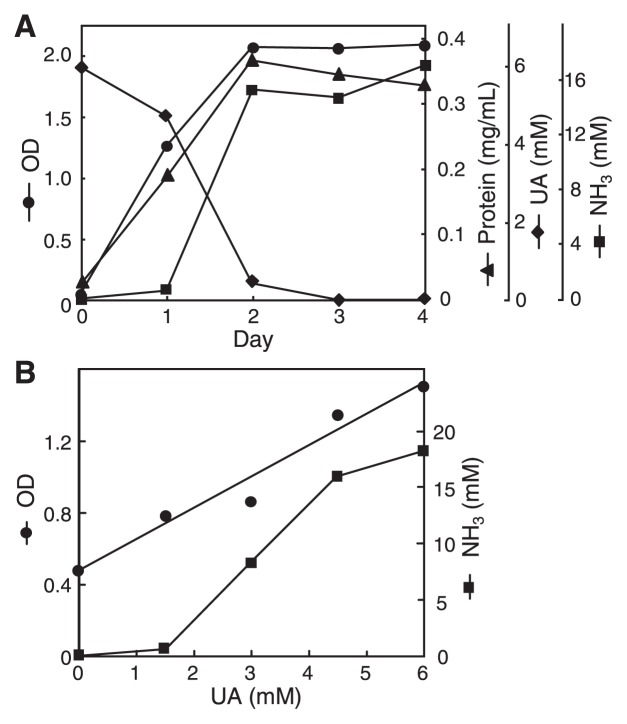
Time course (A) and dose effect (B) of utilization of uric acid as a nitrogen source by strain NkU-1. (A) GY medium supplemented with 0.1% UA was used, and cell growth (OD and protein), UA in the culture, and produced NH_3_ were measured at 24-hour intervals. (B) GY medium supplemented with the defined concentration of UA was used and OD, protein content (data not shown), and produced NH_3_ were measured after three days culture. Linear correlations were obtained for OD (*R*^2^=0.96) and protein (*R*^2^=0.90).

Addition of glucose, cellobiose, fructose, lactose, or xylose stimulated the growth of strain NkU-1 in otherwise growth-limiting medium (Y medium), but UA did not, although UA was partially degraded and NH_3_ was produced (Table 3). The result indicated that strain NkU-1 did not utilize UA as a sole carbon and energy source. Strain NkU-1 efficiently utilized UA as a nitrogen source when these carbon sources were supplied (Table 3), and UA served as a good nitrogen source as ammonium chloride and ammonium sulfate (data not shown). UA was completely or largely degraded in the presence of these carbon sources and a large amount of NH_3_ was produced in the medium. Time course and dose-effect analyses (Fig. 2) confirmed the dependence of UA degradation on cell growth. Only a low level of NH_3_ was excreted at the vigorously growing stage of the cells (Day 1) or at a low level of UA supply (1.5 or 3.0 mM) because nitrogen of degraded UA could be first assimilated into growing cells. Taking this amount of nitrogen into consideration (calculated based on the ratio between the increase of protein from Days 0 to 1 and the amount of nitrogen of degraded UA minus excreted NH_3_ at Day 1), entire recovery of UA nitrogen was obtained as excreted NH_3_ at the later stage of cell growth (98% and 106% at Days 3 and 4, respectively); therefore, strain NkU-1 likely produced a stoichiometric amount of NH_3_ by UA degradation (4 mol NH_3_ from 1 mol UA).

In addition to UA, purine, hypoxanthine, guanine, and xanthine, and the corresponding ribonucleotides to the latter three (inosine, guanosine, and xantosine) stimulated the cell growth of strain NkU-1 (OD and protein concentration (mg mL^−1^) ranged from 0.90 to 0.95 and 0.11 to 0.15, respectively, after three days culture) in otherwise growth-limiting, carbon-rich but nitrogen-poor GY medium (OD and protein reached only 0.36 and 0.04, respectively). Allantoin, allantoic acid, and ribose (ribose does not contain any nitrogen) did not increase cell growth (OD and protein (mg mL^−1^) reached less than 0.39 and 0.06, respectively). When ammonium chloride as a positive control was added to GY medium, OD and protein (mg mL^−1^) reached 0.93 and 0.12, respectively. The results suggest that strain NkU-1 utilizes various purines as a nitrogen source.

The cell suspension of strain NkU-1 degraded UA anaerobically but not aerobically, and UA degradation was detected only in the cell suspension prepared with the culture grown in the presence of UA (data not shown). Aerobic UA degradation generally involves uricase (urate oxidase), which needs molecular oxygen for the catalyzed reaction, and produces allantoin and allantoic acid as intermediate compounds (57). The anaerobic UA degradation pathway and the involved enzymes are considered completely different from those of aerobic, as reported in purinolytic clostridia (48).

### Significance of the isolates and symbiotic UA recycling

A unique feature of strain NkU-1 with respect to UA utilization is that it apparently does not use UA as a carbon and energy source, which is in clear contrast to that of previous isolates from the gut of *R. flavipes* and three purinolytic clostridia species; they utilize UA as a carbon and energy as well as nitrogen source and UA increases the cell yield when incorporated into growth-limiting media (41, 42, 49). Because carbohydrates derived from plant matter are rich in the gut environment and strain NkU-1 can utilize many of them, there is less demand for UA as a carbon and energy source. As opposed to the purinolytic clostridia, none of the isolates from termite guts show an absolute requirement of UA for growth. Strain NkU-1 likely utilizes various purines as a nitrogen source as in the cases of purinolytic clostridia species. The previously isolated strain UAD-1 utilizes only UA effectively, whereas the other examined strains display rather broader versatilities of purines (41). Although the limited versatility reasonably relates to the UA-forming ability of the host termites, as discussed previously (41), the ecological significance of the difference of the versatility of purines is unclear.

A common feature of strain NkU-1 with previous isolates from the termite gut is the excretion of NH_3_ as the major product of UA degradation. As discussed previously (41, 44), NH_3_ produced by UA degradation can be cycled back to the host insect tissue directly or indirectly after assimilation by gut microbiota. For the latter scheme, some behaviors of host termites such as proctodeal trophallaxis (the exchange of hindgut content among nestmates), necrophagy, and cannibalism likely play important roles (7, 21, 55).

Cockroaches also stored UA in their fat bodies and utilization of uric acid nitrogen by intracellular symbionts (*Blattabacterium* sp.) has been hypothesized (6). In the genome sequence of the *Blattabacterium* symbiont, however, there is no candidate gene for uricolytic enzymes (47), suggesting that the symbiont does not play a role, at least in the initial step of UA utilization. One of the plausible alternative explanations is the recycling of UA by gut bacteria as in the case of termites. In a shield bug, bacteria localized extracellularly in the lumen of a specialized structure (so-called swollen crypt) in the midgut (15) have been demonstrated to be responsible for uricolysis during a prereproductive non-feeding period of the host, although associated uricase activity is emphasized (18). Therefore, UA recycling by gut bacteria seems to be a general mechanism for the conservation of nitrogen in terrestrial insects, particularly whose natural diets are low in combined nitrogen. Many insects depend on plant materials and they usually suffer from poverty of nitrogen sources. Meanwhile, yeast-like endosymbionts of planthoppers have been demonstrated to be involved in uric acid recycling, although the related yeast-like symbionts of aphids are not (10). More extensive investigations of the nature of symbiotic recycling of UA in insects are ecologically very important.

Because most studies on anaerobic microbial degradation of UA were conducted more than three decades ago, modern molecular studies are anticipated. Approaches using functional key genes such as *nifH*, *nirK*, and *amoA* are clearly powerful tools for studies on nitrogen cycles in certain environments (e.g. 19, 22, 23, 25, 40, 46, 62, 63) as well as in the termite-gut microbial community (20, 28, 29, 34, 36, 59), but anaerobic UA degradation has not been well-defined genetically; no key gene for this process has been identified. Although experiments with stable isotope measuring or labeling (e.g. 24, 55) may be applicable, the isolation and characterization of anaerobic UA-degrading bacteria are of special significance for further physiological, biochemical, and genome studies, as generally discussed for as-yet-uncultured but ecologically important microorganisms (27, 31). The strains isolated in this study will be very useful for these future studies.

## Figures and Tables

**Table 1:** Anaerobic uricolytic bacteria present in the gut of diverse termites

Termite^a^	Body weight (mg termite^−1^)^b^	CFU per gut	% urico lytic	No. of isolates identified	Identified bacterial group^c^		

					*Clostridia*	*Enterobacteriaceae*	Low G+C Gram-positive cocci
*Reticulitermes speratus* (Rs)	2.1	4.2×10^5^	5.0	2^d^	0	2	0
*Coptotermes formosanus* (Cf)	3.5	8.2×10^5^	4.9	1^e^	1	0	0
*Neotermes koshunensis* (Nk)	15.3	2.2×10^6^	48.6	4^e^	4	0	0
*Glyptotermes fuscus* (Gf)	3.6	3.5×10^6^	0.6	3^e^	0	2	1
*Cryptotermes domesticus* (Cd)	5.4	8.5×10^5^	1.2	31	8 (2)	5 (3)	18 (2)
*Hodotermopsis sjoestedti* (Hs)	63.8	8.4×10^6^	0.1	52	52 (2)	0	0
*Odontotermes formosanus* (Of)	2.7	2.1×10^5^	9.5	19	4	15 (3)	0
*Nasutitermes takasagoensis* (Nt)	3.8	1.5×10^6^	2.0	42	39	0	3

a Initials of termite species used for name tag of the isolate are given in parentheses. Rs and Cf are subterranean termites (Rhinotermitidae). Nk, Gf, and Cd: dry-wood termites (Kalotermitidae). Hs: damp-wood termite (Termopsidae). Of and Nt belong to Termitidae; Of: fungus-grower; Nt: wood-feeder. All the termite species were collected in Japan; Rs in Saitama prefecture, Hs on Yakushima island, and Cf, Nk, Gf, Cd, Of, and Nt on Iriomote island.

b Body weights of these termite species were measured in the previous report (36). Based on these values, CFU per mg individual termite was estimated as 0.78–9.72×10^5^.

c Number of species identified within the group is shown in parentheses when multiple species were identified.

d In Rs, anaerobic bacteria were first isolated and, after identification, representatives were examined for the degradation of UA.

e Uricolytic bacteria were first classified by colony morphology, and only a few representatives were identified. In the other termite species (Cd, Hs, Of, and Nt), a colony of the uricolytic bacteria was randomly picked up and classified based on the sequence similarity of 16S rRNA gene.

**Table 2:** Strains representing uricolytic isolates from termite guts and their closest relatives

Representative strain	Termite host (abundance)^a^	Closest relative^b^	Sequence identity^c^
*Clostridia*			
NkU-1 (JCM 10519)	Nk, Of (4/19), Hs (47/52),	*Clostridium saccharolyticum* (cluster XIVa) (Y18185)	98.9%
Cd6 (JCM 10514)^d^	Cd (7/31), Nt (39/43)	*Clostridium sphenoides* (cluster XIVa) (AB075772)	99.9%
Cd13 (JCM 10515)	Cd (1/31)	*Clostridium bifermentans* (cluster XI) (AB075769)	100%
Hs50 (JCM 10522)	Hs (5/52)	*Clostridium sporogenes* (cluster I) (X68189)	99.3%
CfU-1 (JCM 10513)	Cf	*Clostridium subterminale* (cluster I) (AF241844)	100%
*Enterobacteriaceae* (*Gammaproteobacteria*)			
RsN-1 (JCM 17987)^d^	Rs	*Enterobacter amnigenus* (AB004749)	99.3%
GfU-1 (JCM 17988)	Gf	*Enterobacter aerogenes* (AB004750)	98.8%
Cd20b (JCM 17989)	Cd (2/31)	*Enterobacter asburiae* (AB004744)	98.7%
Cd15a (JCM 17990)	Cd (1/31)	*Enterobacter cowanii* (AJ508303)	98.0%
Cd22 (JCM 17991)	Cd (2/31)	*Serratia nematodiphila* (EU036987)	99.9%
Of17 (JCM 17992)^d^	Of (9/19)	*Trabulsiella odontotermitis* (DQ453129)	99.5%
Of6 (JCM 17993)	Of (4/19)	*Citrobacter farmeri* (AF025371)	99.6%
Of24 (JCM 17994)	Of (2/19)	*Cedecea neteri* (AB086230)	99.7%
Low GC Gram positive cocci (*Bacilli*)			
Cd31 (JCM 10523)^d^	Gf, Cd (2/31)	*Lactococcus lactis* subsp. *lactis* (AB100803)	100%
Cd23 (JCM 10521)	Cd (16/31)	*Enterococcus caccae* (AY943820)	99.4%
Nt1 (JCM 10537)	Nt (3/43)	*Staphylococcus epidermidis* (D83363)	99.3%

a Abbreviations of termite hosts are shown in Table 1. Abundance data shown in parentheses for the four termites Hs, Cd, Nt, and Of correspond to the number of allied isolates divided by the number of examined isolates with a slash.

b Accession number of the 16S rRNA gene sequence of type strain used for comparison is shown in parentheses. In *Clostridium* spp., the clostridia cluster defined by Collins *et al.*(5) is also shown. To our knowledge, there is no report of obvious anaerobic UA degradation in these close relatives except for *C. sphenoides* (this study).

c Almost the entire sequence of the amplified fragment was determined and compared in strains NkU-1 and Cd23, and strains in *Enterobacteriaceae*, Only a partial sequence (less than 690 bp) was compared in the other strains.

d Strain Cd6 showed 99.4% sequence identity to clone HsH-8 from the gut of Hs (30). Strain RsN-1 showed 99.6% sequence identity to clone Rs-M74 from Rs (11). Strain Of17 showed 99.2% sequence identity to clone BOf3-07 from Of (52) and 99.6% identity to clone Nt2-100 from Nt (13). Strain Cd31 showed 100% sequence identity to clone RsaP87 from *R. santonensis*(60).

**Table 3:** Utilization of uric acid by strain NkU-1 as a nitrogen source with various carbon sources

Carbon source	Addition of UA	OD	Protein (mg mL^−1^)	NH^3^ produced (mM)	UA remained (mA)
None	−	0.18	0.050	0.11	NT
None	+	0.19	0.078	6.41	4.66
Glucose	−	0.79	0.086	0.59	NT
Glucose	+	1.21	0.170	18.23	0
Cellobiose	−	0.78	0.082	0	NT
Cellobiose	+	1.14	0.147	20.58	0
Fructose	−	0.47	0.064	0	NT
Fructose	+	1.21	0.142	15.29	0
Lactose	−	0.44	NT	0	NT
Lactose	+	0.94	NT	20.29	0.41
Xylose	−	0.39	NT	0.53	NT
Xylose	+	0.70	NT	18.15	1.42

UA was added to 5.95 mM. NT: not tested.
